# Changes in Non-Deamidated versus Deamidated Epitope Targeting and Disease Prediction during the Antibody Response to Gliadin and Transglutaminase of Infants at Risk for Celiac Disease

**DOI:** 10.3390/ijms23052498

**Published:** 2022-02-24

**Authors:** Ádám Diós, Bharani Srinivasan, Judit Gyimesi, Katharina Werkstetter, Rudolf Valenta, Sibylle Koletzko, Ilma R. Korponay-Szabó

**Affiliations:** 1 Department of Pediatrics, Faculty of Medicine, University of Debrecen, 4032 Debrecen, Hungary; dios.adam@med.unideb.hu; 2 Doctoral School of Molecular Cell and Immune Biology, University of Debrecen, 4032 Debrecen, Hungary; 3 Department of Pathophysiology and Allergy Research, Division of Immunopathology, Center for Pathophysiology, Infectiology and Immunology, Medical University of Vienna, 1090 Vienna, Austria; bharani.srinivasan@cshs.org (B.S.); rudolf.valenta@meduniwien.ac.at (R.V.); 4 Research Division of Immunology, Department of Biomedical Sciences, Cedars-Sinai Medical Center, Los Angeles, CA 90048, USA; 5 Celiac Disease Center, Heim Pál National Paediatric Institute, 1089 Budapest, Hungary; gyimesi.gallisz@gmail.com; 6 Dr. von Hauner Children’s Hospital, Department of Pediatrics, University Hospital, LMU Munich, 80337 Munich, Germany; katharina.werkstetter@med.uni-muenchen.de (K.W.); sibylle.koletzko@med.uni-muenchen.de (S.K.); 7 National Research Centre (NRC), Institute of Immunology Federal Medical-Biological Agency (FMBA) of Russia, 115478 Moscow, Russia; 8 Laboratory for Immunopathology, Department of Clinical Immunology and Allergy, Sechenov First Moscow State Medical University, 119435 Moscow, Russia; 9 Karl Landsteiner University of Health Sciences, 3500 Krems, Austria; 10 Department of Pediatrics, Gastroenterology and Nutrition, School of Medicine, Collegium Medicum, University of Warmia and Mazury, 11082 Olsztyn, Poland

**Keywords:** celiac disease, deamidated gliadin peptides, transglutaminase antibody

## Abstract

Celiac disease (CeD) is a conditional autoimmune disorder with T cell-mediated immune response to gluten coupled with antibody production to gliadin and the self-protein tissue transglutaminase (TG2). TG2 contributes to the CeD pathomechanism by deamidating gliadin, thereby generating more immunogenic peptides. Anti-gliadin antibodies may appear before the autoantibody production. The scope of this study was to dissect these early antibody responses by investigating serum samples collected during the PreventCD prospective double-blind study, where infants with high CeD risk were randomized to 200 mg daily gluten intake or placebo from 4 to 6 months of age, followed by frequent blood testing on regular gluten consumption in both groups. After primary gluten intake, children with or without later CeD produced IgA and IgG antibodies which preferentially recognized non-deamidated gliadin peptides. At CeD development with anti-TG2 seroconversion, there was a significant increase in the antibody reaction toward deamidated gliadin peptides (DGP), with maturation in the binding strength for the PEQPFP gamma-gliadin core peptide. The earliest produced autoantibodies targeted TG2’s celiac epitope 2. Our results reveal a qualitative change in the gliadin-directed humoral immune response at the time when anti-TG2 antibodies appear, but anti-DGP antibodies in the absence of anti-TG2 antibodies are not disease-predictive.

## 1. Introduction

Celiac disease is a T cell-mediated enteropathy with autoimmune features induced by the consumption of gluten-containing cereals in genetically susceptible subjects. The hallmark of the disease is the specific autoantibody production against the self-protein tissue transglutaminase (TG2) coupled with the adaptive anti-gliadin immune response [1]. The disease-inducing gluten prolamins are found in wheat (gliadins and glutenins), barley (hordeins) and rye (secalins), which contain proline and glutamine-rich sequence motifs resistant to human intestinal protease processing [2]; hence, intact gluten peptides can traverse the intestinal barrier and could be presented by the disease-associated HLA-DQ2 or DQ8 molecules [3]. The presence of the predisposing HLA-DQ molecules is indispensable for CeD development; however, it is not sufficient *per se*, and additional predisposing non-HLA genetic variations are required [4].

The multifunctional enzyme TG2 can mediate deamidation of certain glutamine residues in the prolamin peptides or cross-link them to lysine donors. Both these posttranslational modifications may contribute to CeD pathogenesis [5]. The recognition motif for the TG2-mediated deamidation is QXP [6], which is present in the majority of the gliadin sequences recognized by T and B cells [7,8]. Since deamidation of these prolamin peptides further improves their fitting to the antigen-binding pocket of the CeD-associated HLA-DQ molecules [9], this process may exponentially amplify the immune response.

Among gluten prolamins, gliadins are the most potent immunogens for CeD patients, as the majority of patient-derived T and B cell clones can be activated by gliadin peptides [8,10]. Multiple studies revealed that in the course of the humoral adaptive immune response, QPQQPFP and its deamidated QPEQPFP γ-gliadin-derived sequence are the most targeted motifs by antibodies [11,12,13].

In contrast, anti-TG2 autoantibodies target more complex surface epitopes which are clustered on the N-terminal part of the enzyme. Among others, two major epitopes have been described: the discontinuous epitope 1 comprising amino acids K30, R116 and H134 [14], and the conformational multi-domain epitope 2 with anchor residues R19, E153 and M659 [15].

It was reported earlier that the production of gliadin antibodies precedes both the clinical disease manifestation and the production of TG2-specific autoantibodies [16,17]. However, as in the case of many other disorders, we are missing information about the preceding immune response which will predict the loss of tolerance to gluten and lead to the autoimmune CeD process. In the majority of prospective clinical studies, at-risk subjects were not extensively tested in early age prior to the symptoms, and most screening studies focused on the measurement of TG2 autoantibodies to detect transient or persistent autoimmunity, and manifest CeD [18,19].

The international PreventCD study [20] offers a unique opportunity to prospectively follow the development of the humoral immune reaction in persons at risk for CeD from the first gluten intake and look for differences between those who develop CeD and those who remain healthy. As the name indicates, the PreventCD study aimed at prevention of CeD by administration of small doses of gluten to children at genetic risk for CeD. The children were randomized to receive 200 mg of gluten daily for 8 weeks from week 17 of age (4 months, Group A), or a placebo (Group B). With the help of prospectively collected serum samples, the scope of the present study was to simultaneously evaluate the antibody response to the most relevant antigens in CeD, namely, non-deamidated gliadin peptides (NGP), deamidated gliadin peptides (DGP) and TG2, and its evolution over time in children consuming gluten regularly from foods in both groups after 6 months of age. Furthermore, our aim was to monitor and compare the epitope targeting and binding strength of the early gliadin-specific antibodies and to investigate the epitope specificity of the emerging anti-TG2 autoantibodies.

According to our study, infants at risk for CeD generate a vigorous early antibody response to both NGP and DGP, of which the NGP-directed reaction is more pronounced at an early age. Antibody binding to deamidated antigens increases by the time of diagnosis coupled with the production of TG2-directed autoantibodies with celiac epitope specificity. However, gliadin-specific antibodies in the absence of anti-TG2 antibody positivity do not indicate a celiac immune response.

## 2. Results

### 2.1. Infants at Risk of CeD Responded with Anti-Gliadin Antibody Production to Gluten Intake

**Figure 1:**
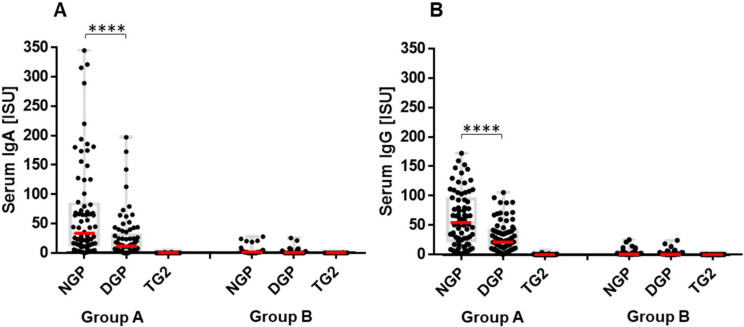
Serum IgA (**A**) and IgG (**B**) antibody production in response to primary gluten ingestion at the end of the 8-week intervention period of either 200 mg gluten (Group A, *n* = 74) or placebo (Group B, *n* = 48). Serum antibody bindings to non-deamidated (NGP) or deamidated (DGP) gliadin peptides, and to transglutaminase 2 (TG2) were measured on protein microarray chip by monoclonal anti-human IgA or IgG labeled with Alexa Fluor 546 as secondary antibodies. ISU denotes detected fluorescent intensity proportional to antibody concentration in serum. Antibody concentrations are presented with medians (red dashes) and interquartile ranges (gray boxes). *p* < 0.05 was considered as significant by one-way ANOVA analysis. **** *p* < 0.0001.

In the study, infants at risk of CeD starting at 4 months of age received either gluten (Group A) or a placebo (Group B) for 8 weeks. At 6 months of age, infants in Group A developed serum IgA (Figure 1A) and IgG (Figure 1B) antibodies to both NGP and DGP, but not against TG2. Children in the placebo arm (Group B) remained negative for all these antibodies. IgA antibody production against gliadins indicates the infant’s own adaptive immune response to the ingested food antigens, since this isotype is not transferred transplacentally from the mother to the infant or absorbed from breast milk [21].

### 2.2. The Early Antibody Response Is Directed Preferentially to NGP, while at the Time of CeD Development, Antibody Response Increases for DGP

**Figure 2:**
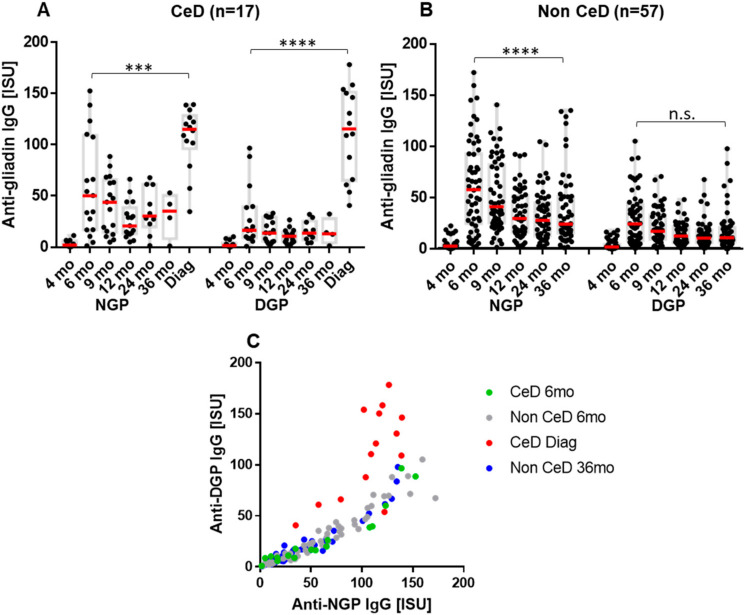
Anti-gliadin IgG antibody response over time in Group A of at-risk infants who developed celiac disease (CeD) (**A**, *n* = 17) or remained disease-free (**B**, *n* = 57). The early immune response after the start of gluten intake is dominated by antibodies against non-deamidated (NGP) over deamidated (DGP) gliadins with a high correlation of individual NGP and DGP antibody levels, while. at the time of CeD diagnosis, there was a shift towards a preference for DGP (**C**). Serum IgG antibody binding was measured on protein microarray chip by monoclonal anti-human IgG, labeled with Alexa Fluor 546 as secondary antibodies. Dashes in red indicate medians, gray boxes indicate interquartile ranges. mo, months of age (in panel A age without manifest CeD); Diag, time of CeD diagnosis (2 to 5.5 years of age, median 3). ISU denotes detected fluorescent intensity proportional to antibody concentration in serum. *p* < 0.05 was considered as significant by one-way ANOVA analysis. *** *p* < 0.001, **** *p* < 0.0001.

In Group A, serum antibody response measured against NGP and DGP using a custom MeDALL protein microarray was compared among children who later developed CeD (*n* = 17, Figure 2A) and those who did not develop CeD (*n* = 57, Figure 2B). The IgG antibody response, as reflected in serum concentrations, showed two peaks in children who developed CeD, compared to only one peak in children without CeD matched for age. The first peak was observed in Group A at the age of 6 months, i.e., in the serum sample taken 2 months after starting intake of low amounts of gluten. The second, much stronger peak (*p* < 0.001 compared to 6 months, Figure 2A) was seen at the time of biopsy-proven CeD diagnosis, which occurred at the age of 2 to 5.5 years (median age 3 years). No difference was found in the early response in participants who later developed CeD compared to those who remained disease-free. Antibodies preferred to recognize NGP over DGP at 6 months of age (*p* < 0.0001, Figure 1) and also during the period of time without disease in the follow-up. Significant increases in serum antibody concentrations to DGP were detected in children who developed CeD at the time of diagnosis (Δ IgG_DGP_ +95.62 ISU (SD ± 45.24), *p* < 0.0001, compared to the last follow-up sample before anti-TG2 seroconversion (Supplementary Figure S1A). The Δ IgG_DGP_ was significantly higher than Δ IgG_NGP_ (+71.09 ISU, SD ± 32.8) for the same samples, *p* = 0.023. In the same comparison, IgG DGP antibodies increased 6.8 times, while NGP antibodies increased only 3.0 times (Supplementary Figure S1A). This shift toward DGP recognition is also demonstrated in the correlation of individual IgG NGP and DGP serum antibody levels, which differed from the non-CeD children of similar age and also from the samples collected at 6 months of age (Figure 2C).

IgA antibodies of children in Group A displayed a similar trend with comparable Δ IgA increase for DGP (+75.06 ISU, SD ± 92.56) and NGP (+79.38 ISU, SD ± 170.6) in mean serum concentrations (not significant, Supplementary Figure S1A). However, the increase was 4.62 times for IgA DGP but only 2.27 times for NGP compared to the last sample before anti-TG2 seroconversion, indicating a preferential increase for DGP also in IgA class at the time of CeD diagnosis (Supplementary Figure S1A).

**Figure 3:**
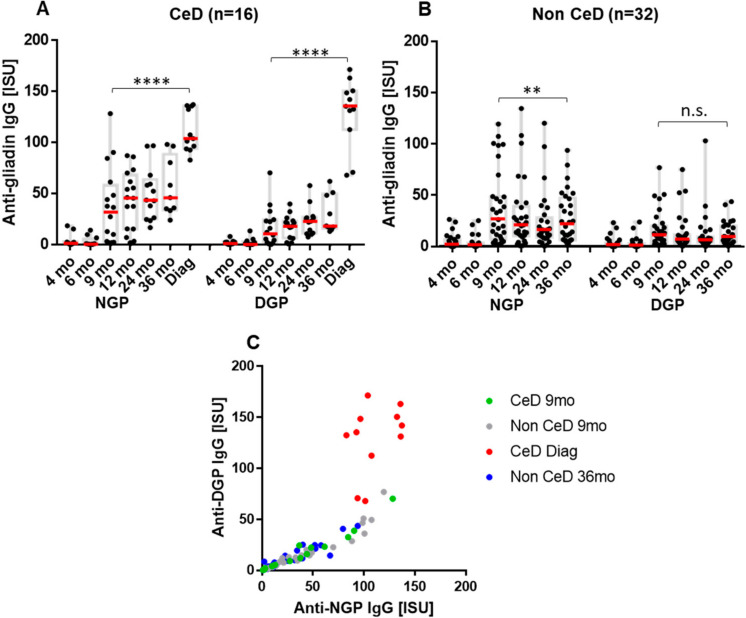
Anti-gliadin IgG antibody response over time in the placebo arm (Group B) of infants who developed celiac disease (CeD) (**A**, *n* = 16) or remained disease-free (**B**, *n* = 32). The immune response after the start of eating gluten-containing foods from the age of 6 months is dominated by antibodies against non-deamidated (NGP) over deamidated (DGP) gliadins, while correlation of individual NGP and DGP antibody levels (**C**) at the time of CeD diagnosis showed preference for DGP. Serum IgG antibody binding was measured on protein microarray chip by monoclonal anti-human IgG, labeled with Alexa Fluor 546 as secondary antibodies. Dashes in red indicate medians, gray boxes indicate interquartile ranges. mo, months of age (in panel A age without manifest CeD); Diag, the time of CeD diagnosis (2 to 5.5 years of age, median 3). ISU denotes detected fluorescent intensity proportional to antibody concentration in serum. *p* < 0.05 was considered as significant by one-way ANOVA analysis. ** *p* < 0.01, **** *p* < 0.0001.

In Group B, we observed a similar pattern; serum antibodies to NGP and DGP appeared at 9 months of age with no differences between those developing later CeD (*n* = 16, Figure 3A) and those without (*n* = 32, Figure 3B). Binding at this time was preferred toward NGP over DGP and shifted to DGP (Figure 3C) at the time of CeD diagnosis (Δ IgG_DGP_ +109.3 ISU, SD ± 33.55 versus Δ IgG_NGP_ +69.54 ISU, SD ± 29.28, *p* = 0.0042, Δ IgA_DGP_ +50.03 ISU, SD ± 44.84 versus Δ IgA_NGP_ +52.46 ISU, SD ± 75.0, p:not significant, respectively). The increase was 6.38 times in IgG DGP antibodies, 2.68 times in IgG NGP, 5.72 times in IgA DGP and 2.5 times in IgA NGP also in Group B (Supplementary Figure S1B).

### 2.3. Increase in Binding Strength of Anti-Gliadin Antibodies for the Deamidated PEQPFP Core Epitope at the Manifestation of CeD

In order to evaluate whether the antibodies produced in response to the primary gluten intake targeted the major disease relevant epitopes and to compare their binding strength with the antibodies produced at the time of CeD diagnosis, we performed label-free molecular interaction studies with single-epitope-bearing synthetic gliadin peptides in bio-layer interferometry. We individually affinity-purified anti-gliadin antibodies from serum samples collected at 6 months of age from subjects who did not develop CeD (*n* = 3) or who later developed CeD (*n* = 3), as well as at the time of CeD diagnosis (*n* = 4). We measured the equilibrium dissociation constant (K_D_) separately to the non-deamidated PLQPQQPFP (γGlia_Q), deamidated PLQPEQPFP (γGlia_E) and shorter deamidated PEQPFP (γGlia_sh) peptides, each offering only one single binding site for the antibodies.

**Figure 4:**
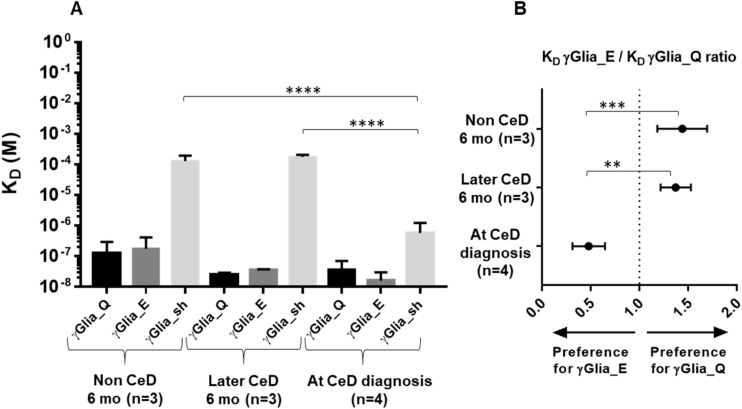
Increase in binding strength toward the PEQPFP core motif of γ-gliadin. Binding strength (**A**) of anti-gliadin antibodies produced at 6 months of age (*n* = 6) and at the time of celiac disease (CeD) diagnosis (*n* = 4) of Group A children was measured by real-time label-free biolayer interferometry to 3 μM of biotinylated γGlia_Q, γGlia_E or γGlia_sh peptides coated to Streptavidin biosensors, respectively. Antibodies were added in 240, 120, 60 and 30 nM concentrations and equilibrium dissociation constants (K*_D_*) were calculated by the BLItz Pro 1.2.1.5 software. (**B**) The ratios of γGlia_E/γGlia_Q K*_D_* values are shown in the same groups. mo, months of age. Bars represent means with SD. *p* < 0.05 was considered as significant by one-way ANOVA analysis. ** *p* < 0.01 *** *p* < 0.001, **** *p* < 0.0001.

At the age of 6 months, infants who later developed CeD and who did not develop CeD both produced antibodies that displayed considerable binding strength to γGlia_Q (mean K_D_ 2.5 × *10^−8^ M and 1.2 × 10^−7^ M, respectively) as well as to γGlia_E peptides (mean K_D_ 3.5 × 10^−8^ M and 1.7 × 10^−7^ M, respectively), which indicates that infants’ early antibody response targeted the major celiac-relevant gliadin epitope PLQPQ/EQPFP (Figure 4A). However, antibodies produced at this age by both these groups did not bind in a relevant manner to the γGlia_sh PEQPFF peptide (mean K_D_ 1.3 × 10^−4^ M and 1.8 × 10^−4^ M, respectively).

In contrast to 6 months, the antibodies produced by the children at the time of CeD diagnosis showed significant increases in binding strength for the γGlia_sh core epitope PEQPFP (mean K_D_ 5.8 × 10^−7^ M, *p* < 0.0001 versus at 6 months), besides having similarly high binding strength as at the age of 6 months to γGlia_Q and γGlia_E (mean K_D_ 3.6 × 10^−8^ M and 1.6 × 10^−8^ M).

The anti-gliadin antibodies produced at the age of 6 months tended to have slightly higher binding strength (i.e., having a lower K_D_) for the non-deamidated (γGlia_Q) peptide, whereas at the time of CeD diagnosis, the binding strength was higher for the deamidated (γGlia_E) peptide (Figure 4A), but the difference in mean K_D_ values did not reach statistical significance. However, when we calculated the K_D_ ratios γGlia_E/γGlia_Q in individual subjects, and thus the relative binding strength for the deamidated versus non-deamidated antigen within the same person, the mean K_D_ ratio of 1.38 at 6 months decreased to a mean K_D_ ratio of 0.48 at the time of CeD diagnosis, and this lower ratio indicates a significant increase (*p* < 0.01) in the relative binding strength for the deamidated peptide (Figure 4B).

### 2.4. Presence of Gliadin-Specific Antibodies in the Absence of Anti-TG2 Positivity Does Not Predict Having or Later Developing CeD

We observed that antibodies to NGP or DGP are common in CeD-risk infants who consume gluten. We performed receiver operating characteristics (ROC) analysis at different cut-off concentrations (Supplementary Table S1) to examine whether we can discriminate CeD and non-CeD cases based on the NGP- or DGP-directed antibody response prior to and at the time of emerging anti-TG2 antibody positivity.

**Figure 5:**
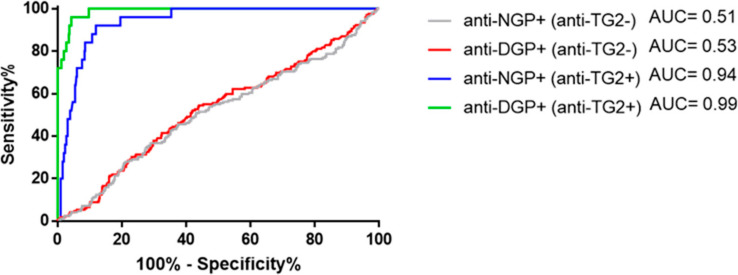
Antibodies to non-deamidated (NGP) or deamidated (DGP) gliadin peptides have no prediction for later celiac disease in the absence of anti-transglutaminase antibodies. Receiver operating characteristic curves for serum IgG antibody-positive samples at any age with gluten consumption to NGP or DGP in the absence or presence of concomitant IgA transglutaminase antibody (anti-TG2) positivity (>3.9 ISU). AUC, area under the curve. The graph is based on values of 562 samples obtained from the 122 children during gluten consumption.

The ROC curves for IgG antibodies (Figure 5) resulted in low area under the curve (AUC) values of 0.51 for NGP antibodies and 0.53 for DGP antibodies in the absence of anti-TG2 antibody positivity, and could not distinguish, at any NGP or DGP concentrations, subjects who later developed CeD or did not. However, when the ROC analysis was performed for NGP/anti-TG2 or DGP/anti-TG2 antibody double-positive samples registered at any age with gluten consumption, AUC improved to 0.94 and 0.99 for NGP and DGP, respectively. IgA anti-gliadin antibodies had the same characteristics, with slightly inferior AUC values (Supplementary Figure S2, Supplementary Table S1). Taken together, in the absence of anti-TG2 positivity, neither the early gliadin antibody response after gluten introduction, nor any anti-gliadin antibody positivity at later age was predictive of having or later developing CeD.

### 2.5. The Anti-TG2 Immune Reaction Predominantly Targets TG2’s Celiac Epitope 2

During the clinical follow-up, 33/122 (27%) children were diagnosed with CeD by duodenal biopsies at a median age of 3 years (range 2–5.5). In all these children, anti-TG2 antibodies were detected by clinical tests at diagnosis. Serum samples at diagnosis were available for the present measurements from 24 of the 33 CeD children, because CeD manifested in the other nine children after the inclusion of serum samples for the MeDALL analysis.

**Figure 6:**
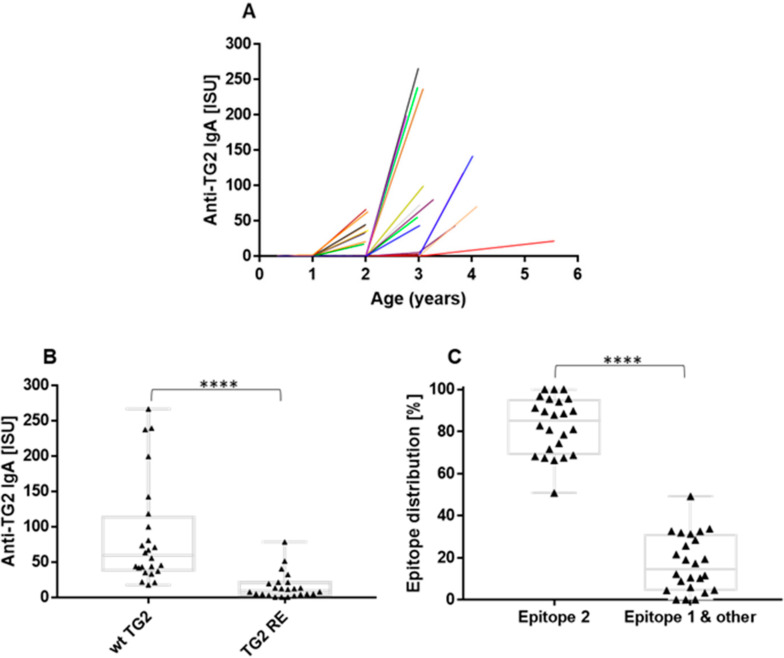
Appearance and epitope specificity of anti-transglutaminase (TG2) antibodies of celiac disease (CeD) patients. (**A**) Serum IgA binding to wild-type TG2 was monitored from the age of 4 months to 5.5 years in the cohort of risk patients (*n* = 122), with most CeD patients seroconverting prior to 3 years of age. Colored lines represent individual patients. (**B**) Bindings to wild-type TG2 and epitope 2-depleted double mutant TG2 RE (R19S, E153S) were measured by protein microarray chip using monoclonal anti-human IgA labeled with Alexa Fluor 546 secondary antibodies at the time of CeD diagnosis (*n* = 24). (**C**) Distribution of epitope specificity calculated from the values of panel B where the binding to wild-type TG2 was set to 100%. ISU denotes detected fluorescent intensity proportional to antibody concentration, boxes indicate interquartile ranges with median. *p* < 0.05 was considered as significant by two-tailed paired *t*-test analysis, **** *p* < 0.0001.

Monitoring the appearance of anti-TG2 antibodies in the serum, elevated IgA antibodies to TG2 were detected in all evaluable 24 CeD cases by the chip method, but only in the samples collected at the time of CeD diagnosis (Figure 6A). IgG anti-TG2 antibodies were detectable in 20/24 CeD cases in low concentrations (data not shown). Elevated levels of antibodies to NGP and DGP occurred in all CeD cases at diagnosis, but their serum concentrations did not correlate with those of anti-TG2 antibodies based on Pearson’s test (TG2 versus NGP r = 0.21 and TG2 versus DGP r = 0.16 for IgA, respectively; Supplementary Figure S3).

The first appearing anti-TG2 autoantibodies were examined for their epitope specificity by utilizing a double-mutant TG2 enzyme (TG2 RE). In TG2 RE, the key anchor residues of celiac epitope 2 are replaced by serine (R19S, E153S), which abolishes epitope 2-specific anti-TG2 antibody binding [15]. Serum anti-TG2 IgA binding to the TG2 RE mutant significantly diminished compared with the binding to the wild-type TG2 (Figure 6B), indicating that the majority of the autoantibodies produced by manifest CeD patients target the epitope 2 surface of human TG2, and/or that their binding is reduced by alterations of the TG2 structure by the mutations. Antibody binding to epitope 1 and other surfaces accounted for17.23% (mean) of the binding to wild type TG2 (Figure 6C).

## 3. Discussion

It is well known that the anti-gliadin immune reaction can be detected prior to and at the time of CeD diagnosis. However, we have limited knowledge about the characteristics of the adaptive immunity toward NGP and DGP after starting gluten intake in children who later develop CeD compared to those who remain disease-free. In addition, little is known about the evolution of anti-NGP and anti-DGP reactivity and its usefulness for disease prediction before the onset of autoimmunity indicated by positive TG2 antibodies.

We used the unique advantage of the PreventCD study, which provided equalized dosage of the early-life gluten intake and extensive follow-up of anti-gliadin and anti-TG2 seroconversion after the prospective randomized dietary intervention in children from celiac families harboring HLA-DQ2 or/and DQ8 risk alleles. The defined low dosage during the 8-week intervention period versus placebo with similar increasing gluten consumption of the groups thereafter enabled us to reliably compare the immune response to gluten from 4 or 6 months of age, as well as the accurate and early detection of disease manifestation.

To quantitatively investigate the humoral adaptive immune response, we applied a custom-prepared chip containing recombinant DGP, NGP, wild-type human TG2 and epitope 2-depleted mutant TG2 antigens. While the clinical ELISA/ELIA assays are calibrated for the low range of antibody concentrations to achieve the best possible sensitivity in clinical care and thus signal saturation is common, the fluorescent detection in the chip system allows antibody quantitation in a broad linear range and makes quantitative changes more readable.

To our knowledge, this is the first time the simultaneous follow-up of both NGP and DGP antibody response in infants at risk of CeD and determination of the binding strength of the produced antibodies has been performed.

The results of the German and Hungarian participants in the PreventCD study reveal that the gliadin-specific antibodies produced in response to early-age gluten exposure preferentially recognize non-deamidated gliadins over the deamidated ones, which is in line with our previous results [22]. The positivity for both IgG and IgA class antibodies suggests that antibodies have undergone isotype switching, confirming that the infants responded to the ingested gluten with an active immune process. Furthermore, we could also show that this early anti-gliadin immune response targets a major disease-relevant gliadin epitope (QPQQPFP/QPEQPFP) irrespective of the future development of CeD.

Compared to the early immune response, gliadin-specific antibodies displayed more intense reactions toward the deamidated gliadins at the time of CeD diagnosis. Similar changes in binding preference did not occur at matched ages in the children who did not develop manifest CeD, which suggests the active role of TG2 and its deamidation activity in disease progression.

Biolayer interferometry indicated a high binding strength toward the immunodominant QPQQPFP/QPEQPFP epitope for both the anti-gliadin antibodies produced by children with manifest CeD and as the early immune response to gluten. This observation is in line with results of Snir et al. [23], who described stereotypic antibody response to gliadins, revealing that in CeD patients, gliadin-specific B cells undergo only modest somatic mutation and mostly have germline-encoded residues with inherent recognition ability for gliadin peptides. However, only antibodies produced at the time of the diagnosis of CeD, as inferred in our experiments, had high binding strength toward the short deamidated PEQPFP γ-gliadin motif, indicating an affinity maturation during CeD development. Although K_D_ values do not represent all aspects of the molecular interaction (avidity) of polyclonal antibodies, they were able to reveal improved binding ability of the antibodies during disease progression.

There was also a difference in the antibody binding strength towards the non-deamidated (γGlia_Q) versus deamidated (γGlia_E) gliadin epitope. The binding strength for the deamidated peptide increased significantly (i.e., K_D_ decreased) at the time of CeD diagnosis. This could be statistically demonstrated by evaluating the γGlia_E/γGlia_Q K_D_ ratios for the antibodies within the same subject, even though simple comparison of γGlia_E K_D_ and γGlia_Q K_D_ mean values did not reach significant values. The lack of significance may be related to the considerable differences in antibody avidity/affinity between the subjects, but probably also due to the small number of cases in each group, as only a few serum samples contained high enough amounts of anti-gliadin antibodies and were available in sufficient volumes for affinity purification. Taken together, these results indicate that during CeD development, there is not only an increase in the production of DGP-targeted antibodies, as reflected in their increasing serum concentrations, which has been shown in earlier studies [22], but also an increase in DGP-binding strength of the anti-gliadin antibodies compared to NGP.

The anti-gliadin immune reaction either to NGP or DGP was not predictive on its own of the diagnosis of CeD at any age prior to the development of autoantibodies; hence, children who developed CeD could not be distinguished by ROC analyses from those who remained healthy. Our results prove that anti-gliadin antibodies might be present well before the appearance of anti-TG2 antibodies in the serum, but at this stage do not have a diagnostic significance and are not indicative of CeD, and therefore should not be used as diagnostic tool. Our results confirm that detecting anti-TG2 antibodies is the first-line reliable test for CeD diagnosis.

The serum concentrations of anti-TG2 IgA antibodies were not in linear correlation with those of anti-NGP or DGP. This might be related to the different mechanisms of their triggering and different isotypes. Furthermore, measuring the level of anti-TG2 IgA in serum might underestimate the total amounts of anti-TG2 produced, since these antibodies also deposit in different tissues [24].

We investigated the epitope specificity of the produced anti-TG2 antibodies by applying a double mutant TG2 (R19S, E153S) devoid of epitope 2 [15] and proved that the vast majority of the emerging autoantibodies produced by CeD patients that appear in serum target the celiac epitope 2 on the N-terminal surface of TG2. The histology results of the subsequently performed intestinal biopsies showed that the presence of epitope 2-specific TG2 antibodies was associated with villous atrophy regardless of age.

Despite the regular gluten intake and the continuous presence of TG2 protein in the extracellular matrix, it seems that TG2 as an autoantigen and deamidated gliadin epitopes come into the crosshairs of the immune system only after at least several months of gluten intake, since the first TG2 positivity was observed in this dataset beyond 12 months of age. The preferential recognition of NGP after starting gluten intake might mean that availability of DGP is less at the early (pre-disease) immune response, and that deamidation becomes frequent only at the onset of pathologic responses by cellular stress or other mechanisms. It has been reported by multiple studies that the cellular stress induced by gliadin might eventually lead to TG2 activation and further cascade of innate immune and inflammatory responses [25,26]. Although the highest incidence of seroconversion to positive autoantibodies and CeD manifestation with or without symptoms occurs between 2 and 3 years of age [27], this process may be delayed in predisposed persons even for decades on a regular gluten-containing diet. This indicates that other trigger factors (e.g., intestinal infections, changes in the microbiome, etc.) might also be necessary for a pathologic activation of the TG2 enzyme. It is of great interest to see what directs TG2’s activity toward the gliadin peptides and whether deamidase or cross-linking activity is making a change in CeD pathogenesis. It has been shown in in vitro studies that TG2 can covalently cross-link gliadins to itself [28,29], leading to a possible hapten carrier formation. However, the in vivo formation of such a complex has not yet definitively been proven, and a recent study suggested that the gluten–TG2 complex might be rather a temporary enzyme–substrate intermediate in in vivo conditions [30]. This led us to speculate that enhanced deamidation of gliadin might induce pathogenic changes; however, the role of TG2 as a pander and a victim remains still elusive.

For a more comprehensive picture on the anti-gliadin response of CeD and non-CeD subjects at the pre-manifest phase, the gliadin-specific T cells would be of great interest to examine, but this was beyond the scope of this study. A recent study of the Italian PreventCD cohort demonstrated that gliadin-reactive T cells could be detected in the intestine of TG2 seronegative children with anti-gliadin antibody positivity and normal villus architecture [31]. This observation is in line with our results that the presence of high-affinity, gliadin-specific serum antibodies in non-CeD infants might be associated with ongoing T cell-mediated immune reaction in the small intestine. However, the relationship between the development of CeD and the presence of gliadin-reactive T cells in at-risk children with normal mucosa is not yet established. Furthermore, the same study also pointed out that gliadin-reactive T cells present in the duodenum at the time of CeD diagnosis display more intense interferon-gamma production in response to deamidated gliadins, while non-CeD children with normal mucosa respond to native and deamidated gliadins similarly. This is also concordant with our observations, that gliadin-specific serum antibodies tend to target deamidated epitopes at the time of diagnosis.

One other limitation of our study was that the PreventCD study only enrolled and followed HLA-DQ 2- and/or DQ8-positive children; thus, serum samples were not available from this early age from HLA-DQ2- and DQ8-negative subjects and therefore, the normal response to gluten in non-risk people could not be studied.

## 4. Materials and Methods

### 4.1. Participants

This study utilized prospectively collected serum samples from a subgroup of the international double-blind placebo-controlled PreventCD intervention study (registration number: ISRCTN74582487, http://www.preventcd.com, accessed on 20 February 2022) [20]. HLA-DQ2- and/or DQ8-positive newborns having a first-degree family member with diagnosed CeD were randomized to early gluten introduction at 4 months (week 17) of age by administering gluten powder (Group A) or placebo (Group B) for 8 weeks, followed by feeding gluten-containing baby food items in both groups. Group A received 200 mg per day wheat vital gluten (Danone Research BV, Wageningen, The Netherlands), and Group B received placebo (lactose) between the ages of 4 and 6 months in a double-blind placebo-controlled fashion. Infants in both groups received gradually increasing amounts of gluten between 7 and 10 months of age, with no restrictions thereafter. They were followed clinically and by regular blood samplings at the ages of 4, 6, 9, 12, 24 and 36 months. Additional serum samples were taken if symptoms suggestive of CeD occurred and/or at the time of duodenal biopsies in seropositive cases. For this sub-study, 122 children from Germany (*n* = 60) and Hungary (*n* = 62) were included. CeD was confirmed if histopathology showed Marsh III lesion. Enrollment and sample collection were performed upon informed consent from the parents that their child’s serum would be used for different antibody testing related to CeD in different laboratories.

### 4.2. Peptides and Reagents

Synthetic gliadin peptides with N-terminal biotinylation and >95% purity were obtained from GenScript (Leiden, The Netherlands) with the following sequences: SGGPLQPQQPFP (γ-Glia_Q), SGGPLQPEQPFP (γ-Glia_E) and SGGPEQPFP (γ-Glia_sh), where the SGG sequence was a technical linker. These peptides represented the major coeliac immunodominant T/B cell epitope heptapeptide [10,12,22,32] and its deamidated and shortened deamidated forms, respectively. A complex gliadin peptide highly antigenic in clinical testing utilized for antibody purification was a gift from Inova Diagnostics (San Diego, CA, USA).

### 4.3. Microarray Detection of Serum Antibodies

Custom MeDALL microarray chips [33,34] were prepared for this study with deamidated gliadin peptides (DGP) validated in clinical studies by means of the Gliadin DP ELIA kit (Thermo Fisher Scientific, Freiburg, Germany), their non-deamidated counterparts (NGP, both gifts from Thermo Fisher Scientific), wild-type human recombinant transglutaminase 2 (wt TG2) with valine at amino acid position 224 [35] and its R19S-E153S mutant (TG2 RE) at celiac epitope 2 [15]. Antigens were diluted to a final concentration of 0.3 mg/mL in 150 mM sodium phosphate buffer (pH 8.5) containing 0.1% sodium dodecyl sulfate and were spotted robotically in an ordered array onto pre-activated glass slides developed for the covalent attachment of proteins by VBC-GENOMICS, using an Affymetrix 417 Arrayer (Affymetrix, Santa Clara, CA, USA). Then, the chips were washed in TBS-T (10 mM Tris-HCl, 150 mM NaCl, 0.5% Tween-20, pH 8.0) for 15 min, rinsed with double-distilled water (H_2_Odd) and air-dried. Slides were then incubated with 200 µL of patients’ serum diluted 1:5 in TBS-T by using Probe-Clip Incubation Press Seal Chambers (Sigma, St. Louis, MO, USA) for 60–180 min at 37 °C with shaking at 150 rpm. Slides were then rinsed with TBS-T and washed with TBS-T for another 15 min. Bound IgA and IgG antibodies were detected with a 1:1000 diluted mouse monoclonal anti-human IgA and IgG antibodies (Pharmingen, San Diego, CA, USA), respectively, which had been labeled by using the Alexa Fluor 546 protein labeling kit according to the manufacturer’s protocol. Slides were washed five times in TBS-T, rinsed with H2Odd and air-dried. The slides were then scanned by using an Affymetrix 418 scanner (Affymetrix), with the laser power and PMT gain set to 100%. Data were evaluated with the GenePix Software Version 3.0 (Axon Instruments, Union City, CA, USA) and Microsoft Excel. The mean signal of each triplicate determination was considered positive when it was at least five times higher than the signal obtained for spotted human serum albumin.

### 4.4. Affinity Purification of Gliadin-Specific Antibodies from Patient Serum Samples

Gliadin-specific antibodies were purified from selected PreventCD samples with sufficient amounts, applying 300 µg gliadin (Inova) bound to 300 mg CNBr Activated Sepharose 4B (Thermo Scientific, Rockford, IL, USA) by following the manufacturer’s instructions. Patient serum was diluted two times in phosphate-buffered saline (PBS) pH 7.4 + 0.1% Tween 20 and incubated with the antigen-coated resin for 1 h at room temperature. The antibodies were eluted with 5 column volumes of 100 mM glycine pH 2.5 followed by buffer change to PBS with 50K Amicon^®^ Ultra Centrifugal Filters (Merck, Darmstadt, Germany). Protein concentration was determined by Bio-Rad Protein Assay Reagent (Biorad Laboratories, Hercules, CA, USA) using human IgG as a standard.

### 4.5. Determination of the Binding Kinetics of Affinity-Purified Antibodies by Bli System

Kinetic parameters were determined by bio-layer interferometry in the label-free Personal Assay BLItz System (PALL FortéBio, Fremont, CA, USA). Streptavidin biosensors (FortéBio, Fremont, CA, USA) were coated with 3 µM of biotinylated gliadin peptides in PBS +0.1% Tween 20 (PBS-T) buffer. Affinity purified antibodies were diluted to 240, 120, 60 and 30 nM in PBS-T and measured in duplicates in binding assays with 5 min association and 5 min dissociation time. Biosensors were regenerated in 0.5 M phosphoric acid and reused up to ten times. Equilibrium dissociation constants (K_D_) were determined by the BLItz Pro 1.2.1.5 software using the 1:1 kinetic model.

### 4.6. Statistics

Statistical analyses of NGP or DGP serum antibody binding of CeD and non CeD children and comparison of the antibody affinity values were performed using one-way ANOVA, while the anti-TG2 antibody binding of the same samples to wt and mutant TG2 and the changes in serum anti-NGP and anti-DGP levels from the last pre-disease time point to the time of CeD diagnosis were tested by using paired two-tailed *t*-test. Correlations of anti-TG2 and NGP or DGP antibody values were determined by Pearson’s test, while AUC of NGP and DGP antibody values were calculated by ROC analysis. Figures were prepared using GraphPad Prism7 (San Diego, CA, USA).

## 5. Conclusions

In infants and young children at genetic risk for CeD, the early immune response after starting gluten intake is against both NGP and DGP, but appears to preferentially target NGP. We found no difference in antibody concentrations between subjects with or without later development of CeD. Gliadin-specific antibodies reflect the natural history of the immune response to gluten ingestion and display immune maturation. Their presence in the absence of autoantibodies against TG2 is not predictive for later CeD. Our data strongly support the European guidelines for diagnosing CeD [36] that testing for DGP antibodies should not be performed in the primary diagnostic work-up in children, regardless of age.

## Data Availability

The datasets generated and analyzed during the current study are available from the corresponding author on reasonable request.

## Supplementary Materials

The following supporting information can be downloaded at: https://www.mdpi.com/article/10.3390/ijms23052498/s1.
