# Supplementary material for: Changes in Non-Deamidated versus Deamidated Epitope Targeting and Disease Prediction during the Antibody Response to Gliadin and Transglutaminase of Infants at Risk for Celiac Disease
Source: Int J Mol Sci. 2022 Feb 24;23(5):2498. doi: 10.3390/ijms23052498 (PMC8909931; doi:10.3390/ijms23052498)
Supplement: Supplementary file 1 [file ijms-23-02498-s001.zip › ijms-1599336-supplementary/IJMS_supplement Figures.pdf]

Supplementary Material

Changes in non-deamidated versus deamidated epitope targeting and disease prediction during the antibody response to gliadin and transglutaminase of infants at risk for celiac disease

Ádám Diós, Bharani Srinivasan, Judit Gyimesi, Katharina Werkstetter, Rudolf Valenta, Sibylle Koletzko, and Ilma R. Korponay-Szabó

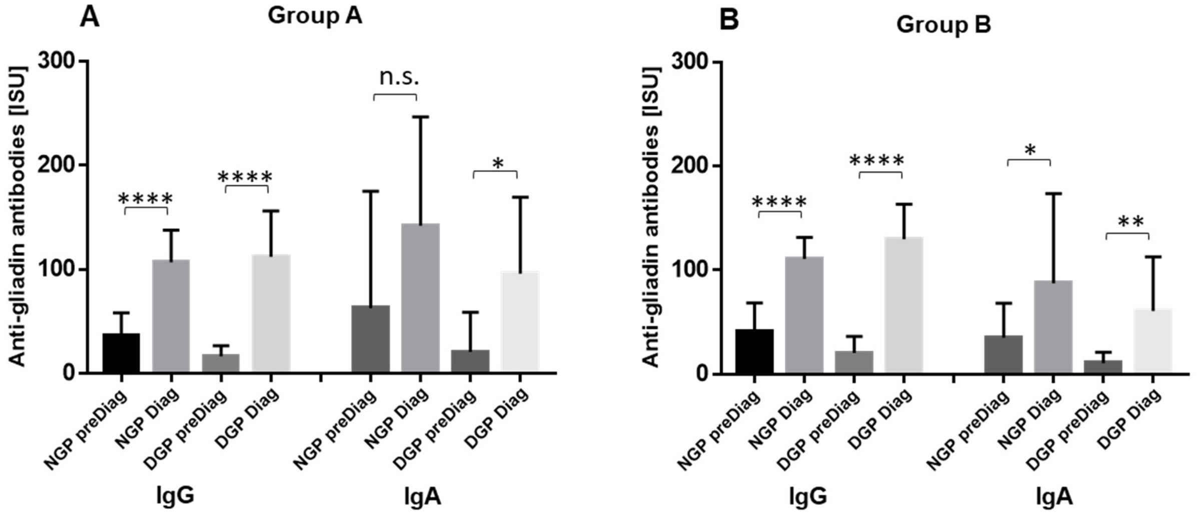

Figure S1. **Increase of anti-gliadin antibody serum levels from the latest visit before seroconversion to transglutaminase antibodies (preDiag) to the time of celiac disease (CeD) diagnosis (Diag) in infants who developed CeD from the early gluten intervention Group A (n=13) and from the placebo arm (Group B, n=11).** Serum IgG and IgA antibody binding were measured to non-deamidated gliadin peptides (NGP) and deamidated gliadin peptides (DGP) on protein microarray chip by monoclonal anti-human IgG or IgA, respectively, labelled with Alexa Fluor 546 as secondary antibodies. Bars represent means with SD. ISU denotes detected fluorescent intensity proportional to antibody concentration in serum. $p < 0.05$ was considered as significant by paired T-test analysis. * $p < 0.05$, ** $p < 0.01$, **** $p < 0.0001$

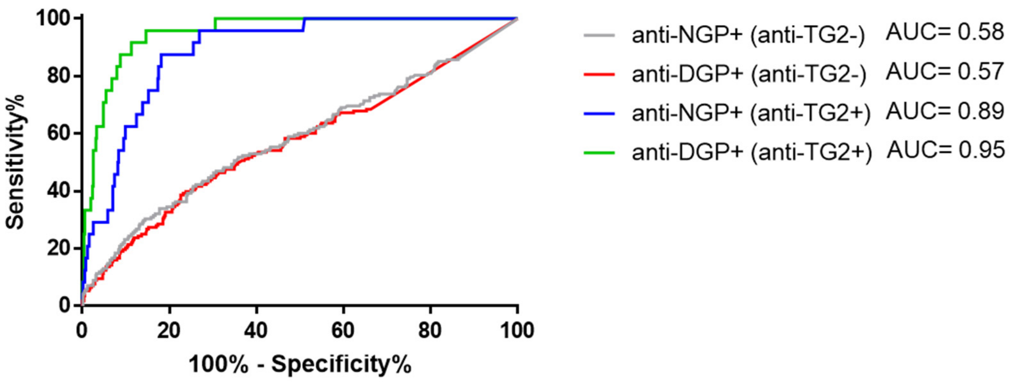

Figure S2. IgA Antibodies to non-deamidated (NGP) or deamidated (DGP) gliadin peptides have no prediction for later celiac disease in the absence of anti-transglutaminase (anti-TG2) antibodies. Receiver operating characteristics curves for serum IgA antibody positive samples at any age with gluten consumption to NGP or DGP in the absence or presence of concomitant IgA anti-TG2 positivity (>3.9 ISU). AUC means area under the curve. The graph is based on values of 562 samples obtained from the 122 children during gluten consumption.

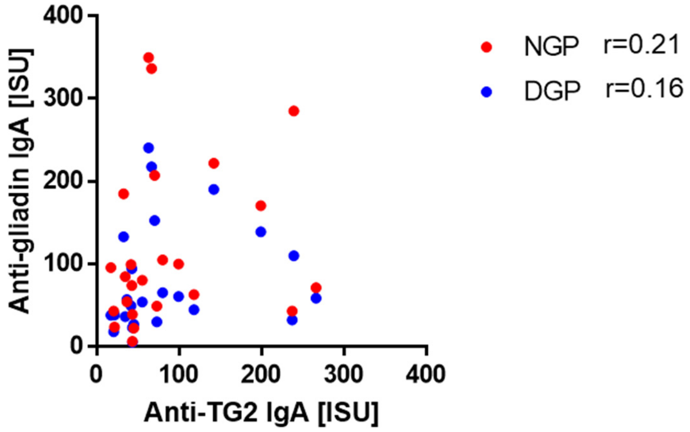

Figure S3. Correlation of IgA anti-gliadin and IgA anti-transglutaminase (TG2) antibody concentrations in serum at the time of celiac disease (CeD) diagnosis. Serum antibody binding of manifest CeD patients (n = 24) was measured to non-deamidated (NGP) and deamidated (DGP) gliadin peptides, and wild type TG2 on protein microarray chip by monoclonal anti-human IgA labelled with Alexa Fluor 546 as secondary antibodies. ISU denotes detected fluorescent intensity proportional to antibody concentration in serum. Correlation coefficient (r) was determined by Pearson correlation test.
